# Cardiovascular magnetic resonance reveals myocardial involvement in patients with active stage of inflammatory bowel disease

**DOI:** 10.1007/s00392-024-02503-5

**Published:** 2024-08-05

**Authors:** Maximilian Fenski, Endri Abazi, Jan Gröschel, Thomas Hadler, Diane Kappelmayer, Frank Kolligs, Claudia Prieto, Rene Botnar, Karl-Philipp Kunze, Jeanette Schulz-Menger

**Affiliations:** 1 Working Group Cardiovascular Magnetic Resonance, Experimental and Clinical Research CenterMax-Delbrück Center for Molecular MedicineDepartment of Cardiology and Nephrology, Charité Medical Faculty, HELIOS Klinikum Berlin Buch, Charité – Universitätsmedizin Berlin Lindenberger Weg 80, 13125 Berlin, Germany; 2 Department of Internal Medicine and Gastroenterology, HELIOS Klinikum Berlin Buch, Berlin, Germany; 3 School of Biomedical Engineering and Imaging Sciences, King’s College London, London, UK; 4 MR Research Collaborations, Siemens Healthcare Limited, Camberley, UK; 5 DZHK (German Center for Cardiovascular Research), Partner Site Berlin, Berlin, Germany; 6 School of Engineering, Pontificia Universidad Católica de Chile, Santiago, Chile; 7 Institute for Biological and Medical Engineering, Pontificia Universidad Católica de Chile, Santiago, Chile

**Keywords:** Crohn’s disease, Ulcerative colitis, Extraintestinal manifestation, Cardiac remodeling, Inflammation

## Abstract

**Background:**

Active inflammatory bowel disease (A-IBD) but not remission (R-IBD) has been associated with an increased risk of cardiovascular death and hospitalization for heart failure.

**Objectives:**

Using cardiovascular magnetic resonance (CMR), this study aims to assess adverse myocardial remodeling in patients with IBD in correlation with disease activity.

**Methods:**

Forty-four IBD patients without cardiovascular disease (24 female, median-age: 39.5 years, 26 A-IBD, 18 R-IBD) and 44 matched healthy volunteers (HV) were prospectively enrolled. The disease stage was determined by endoscopic and patient-reported criteria. Participants underwent CMR for cardiac phenotyping: cine imaging and strain analysis were performed to assess ventricular function. T1 mapping, extracellular volume and late-gadolinium enhanced images were obtained to assess focal and diffuse myocardial fibrosis. Simultaneous T1 and T2 elevation (T1 > 1049.3 ms, T2 > 54 ms) was considered to indicate a myocardial segment was inflamed.

**Results:**

16/44 (16.4%) IBD patients described dyspnea on exertion and 10/44 (22.7%) reported chest pain. A-IBD patients showed impaired ventricular function, indicated by reduced global circumferential and radial strain despite preserved left-ventricular ejection fraction. 16% of all IBD patients had focal fibrosis in a non-ischemic pattern. A-IDB patients had increased markers of diffuse left ventricular fibrosis (T1-values: A-IBD: 1022.0 ± 34.83 ms, R-IBD: 1010.10 ± 32.88 ms, HV: 990.61 ± 29.35 ms, p < .01). Significantly more participants with A-IDB (8/26, 30.8%) had at least one inflamed myocardial segment than patients in remission (0/18) and HV (1/44, 2.3%, p < .01). Markers of diffuse fibrosis correlated with disease activity.

**Conclusion:**

This study, using CMR, provides evidence of myocardial involvement and patterns of adverse left ventricular remodeling in patients with IBD.

**Clinical trial registration:**

ISRCTN30941346

**Graphical abstract:**

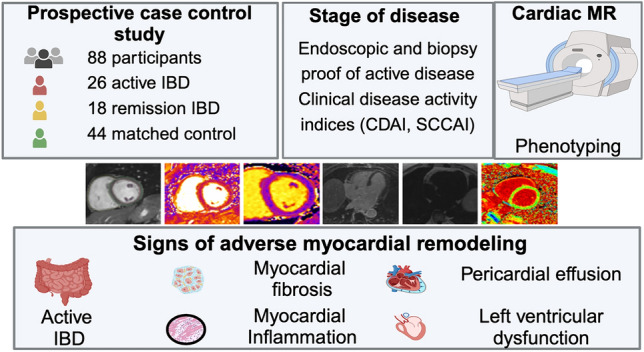

**Supplementary Information:**

The online version contains supplementary material available at 10.1007/s00392-024-02503-5.

## Introduction

Inflammatory bowel disease (IBD), with its main forms Ulcerative colitis (UC) and Crohn’s disease (CD), is an autoinflammatory disorder characterized predominantly by a state of self-directed inflammation in the gastrointestinal tract [1]. Despite intestinal inflammation being the defining characteristic, IBD can be a systemic disorder [2]. Patients with IBD frequently develop extraintestinal manifestations (EIM), that can affect multiple organ systems and can contribute to morbidity [3]. Although not regularly recognized, there is growing evidence for cardiac involvement: IBD-patients were associated with an increased risk of cardiovascular death and heart failure, especially during periods of active disease despite a low prevalence of traditional cardiovascular risk factors [4–6]. The underlying mechanisms are debated [7, 8]. In other systemic immune-mediated diseases, cardiac involvement can be clinically silent and is associated with an adverse outcome [9]. Cardiovascular magnetic resonance (CMR) can detect pathological findings (i.e., myocardial edema, hyperemia and capillary leakage, focal fibrosis and scar) associated with myocardial inflammation and adverse myocardial remodeling [10] and has demonstrated its applicability in identifying heart-involvement in various systemic inflammatory diseases, even in pre-clinical stages [9]. The aim of the study was to gain insight into the extent of myocardial inflammation and adverse left ventricular remodeling in IBD-patients in active and remission stages without known cardiovascular disease by using CMR.

## Methods

**Fig. 1:**
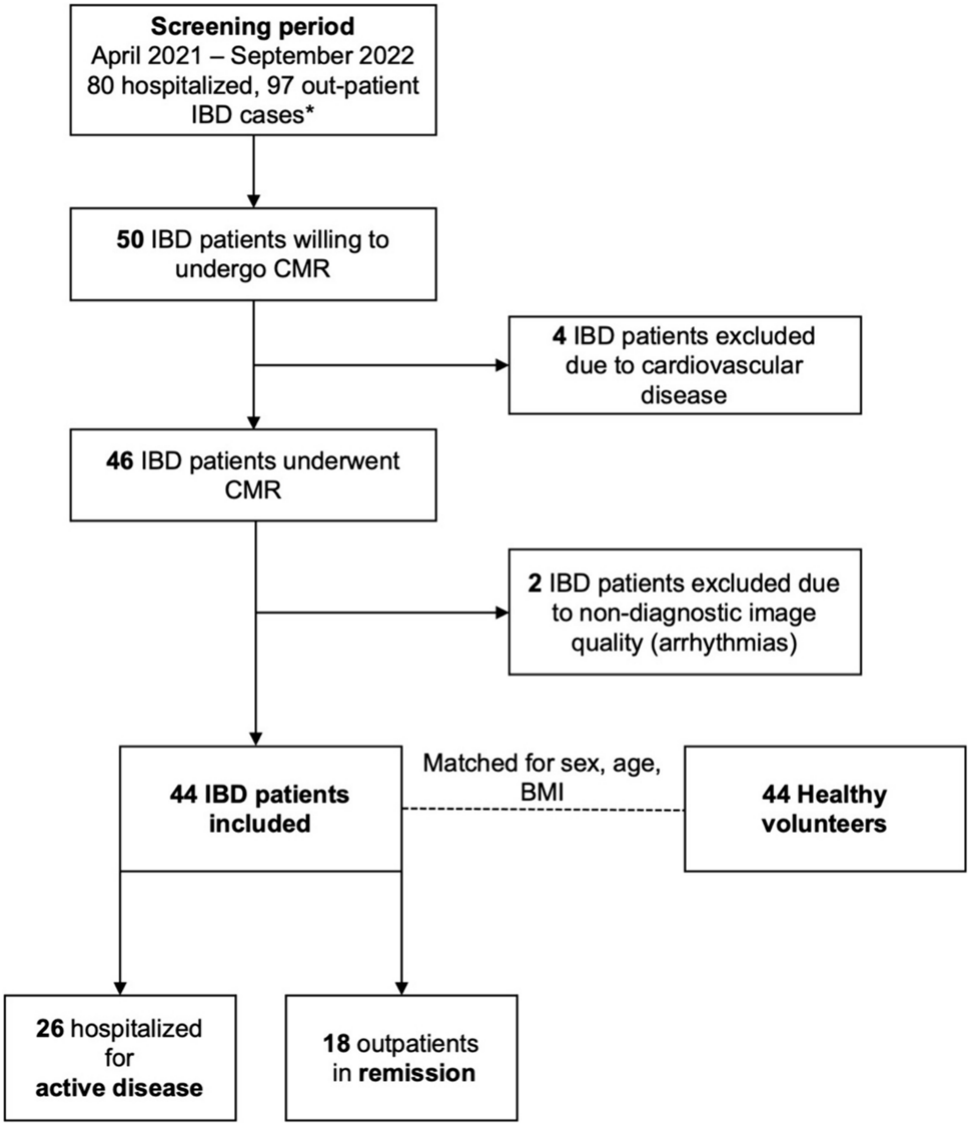
Study flow chart, IBD = Inflammatory bowel disease, * Case number extracted from Hospital Information System, may include multiple presentations of the same patient

This prospective study included non-selected patients with IBD without known cardiovascular disease and healthy volunteers (HV) as a control group. Patients were recruited from one tertiary medical center. The study was performed between April 2021 and September 2022. The strobe diagram in Fig. 1 shows the participants’ enrollment process. Initial IBD diagnosis and disease stage was determined by a board-certified gastroenterologist according to established guidelines [11]. All patients with active disease were hospitalized for acute IBD-flare and had endoscopy and biopsy proof of acute intestinal inflammation. Timing between endoscopy and CMR was median 7 (IQR 1–12) days. Presence of infectious colitis, including Clostridium difficile, was excluded according to current guidelines [11]. Patients in remission were recruited from the out-patient department and had no clinical signs of active IBD. Performance of a repeated invasive endoscopy with biopsy to demonstrate mucosal healing in patients in remission was waived due to ethical concerns. HV were matched one-to-one with IBD patients for age, sex, and body mass index (BMI, kg/m^2^). Age was matched if the difference between an IBD patient and a volunteer was less than 10 years. Participants were matched according to BMI, aiming for ≤ 5 kg/m^2^ differences between both participants, which was achieved in 35/44 matches. The median [interquartile range] differences between the IBD patient and matched volunteer were 1 [0–3] years for age and 3 [2–5] kg/m^2^ for BMI. HVs had no history of cardiovascular disease or cardiac symptoms, did not take systemic medications, had a normal resting ECG, and underwent CMR as control subjects. Exclusion criteria included contraindications to CMR, known cardiovascular disease, renal impairment (estimated glomerular filtration rate < 30 ml/min), known hypersensitivity to gadolinium, and claustrophobia. Pregnant or lactating female subjects were also excluded. All subjects provided written informed consent to participate. Ethical approval was granted by the institutional ethics committee under EA1/198/20.

### Measurement of IBD disease activity

Disease activity scores were assessed prior to the CMR scan using the simple clinical colitis activity index (SCCAI) for patients with UC and the Crohn’s disease activity index (CDAI) for patients with CD. The SCCAI is a validated questionnaire that refers to disease symptoms that occurred during the previous week, assesses symptom severity in six domains and can detect disease activity in remission [12]. A SCCAI < 2 was defined as indicating remission [13]. The CDAI is a widely used tool in clinical trials to determine the severity of illness in patients with CD in eight domains [14]. Scores ≤ 150 are considered to indicate remission and a CDAI > 450 as a marker of severe CD [15]. Individual disease duration was calculated in days from the 1st day of the initial IBD diagnosis (or the 1^st^ day of the month if the exact day was uncertain) to the date of the CMR examination. CMR biomarkers (T1, T2, ECV, strain) were correlated with clinical activity indices and disease duration. SCCAI and CDAI scores were standardized using Z-scoring to include CU and CD patients in one model. The standardized activity index was calculated per patient as:

$$\text{Z}-\text{Score}= \frac{\left(\text{CDAI or SCCAI}\right)-\text{mean }\left(\text{CDAI or SCCAI}\right)}{\text{Standard deviation }\left(\text{CDAI or SCCAI}\right)}$$

### Assessment of heart failure related symptoms

Before undergoing CMR, participants were asked for occurrence of palpitations, angina pectoris or dyspnea as symptoms of heart failure during the last month.

### CMR image acquisition

All participants underwent CMR imaging on a 1.5 Tesla scanner (MAGNETOM AvantoFit, Siemens Healthineers) using ECG gating and a 32-channel surface phased-array coil. For the assessment of biventricular function and dimension, balanced steady-state free precession cine images were acquired in four long-axis views, consisting of a four-, two-, and three-chamber view, as well as a right ventricular (RV) view, and one short-axis (SAX) stack covering both ventricles without a gap. For the assessment of diffuse fibrosis and inflammation, T1-, T2-, and extracellular volume (ECV) fraction mapping images were obtained in three short axis planes (basal, midventricular, and apical). Native T1-mapping was acquired using a motion-corrected modified Look-Locker inversion recovery technique in a 5(3)3 acquisition scheme (i.e., MOLLI 5(3)3). T2-mapping was based on a motion-corrected balanced steady-state free precession sequence. Both were acquired during breath-hold. Synthetic extracellular volume (ECV) fraction was calculated from T1-mapping pre- and post-contrast media application using a prototype sequence.

All sequence parameters correspond to those previously published [16] and are included in the supplement. Combined 3D Late Gadolinium Enhancement and fat/water imaging was obtained 10–15 min after administering 0.2 mmol/kg of contrast media (Gadoteridol, Prohance®, Bracco Imaging) using a free-breathing motion corrected whole heart research sequence with isotropic spatial resolution (1.3 mm^3^) [17]. In patients (n = 9) with a heart rate > 90 beats per minute (bpm), 3D LGE Dixon imaging was replaced by motion-corrected 2D free-breathing LGE imaging with the same slice-positioning as cine images (i.e. covering the whole ventricle without a gap) and a 2D fat/water sequence due to the expected lower image quality impairment of the 2D sequences in patients with arrhythmias or high heart rates [18]. HV underwent a scan protocol including the same sequences and set of parameters but without the application of contrast enhanced imaging.

### CMR image analysis

All CMR images and maps were analyzed off-line by two readers (one with 5 (MF) and one with 2 years of CMR experience (EA)) using CVI42^®^ (version 5.13.0, Circle Cardiovascular Imaging) according to recent post-processing guidelines [19]. The readers were blinded with regard to group allocation and the patients’ disease activity index. Assessment of biventricular function and dimensions was performed on cine SAX images. Left atrial function was evaluated based on cine four- and two-chamber views using a biplanar approach. Myocardial deformation assessment by feature tracking as a marker of left ventricular function was performed to calculate global longitudinal (GLS), circumferential (GCS) and radial (GRS) strain, as described recently [20]. Quantitative mapping analysis was performed with endo- and epicardial border delineation to obtain global and segmental values according to the 17-segment American Heart Association (AHA) model, omitting the apical cap. A 5% contour offset was used to minimize partial volume effects. Apical slices without visible blood pool or thin myocardial walls were excluded. Global, basal, midventricular, and apical T1, T2, and ECV values were calculated as the mean of their corresponding AHA segments after excluding segments with artefacts, focal fibrosis or fat. Areas of myocardial inflammation were defined as AHA segments which had T1 and T2 values exceeding concomitantly the mean + two standard deviations of the global healthy control group according to current guidelines (T1 > 1049.3 ms, T2 > 54.3 ms) [10, 21]. T1 and ECV mapping were used to assess diffuse fibrosis. To differentiate between focal and diffuse fibrosis, AHA segments with focal fibrosis on LGE imaging were excluded from the mapping results as recently published [22]. Presence of pericardial effusion was defined as > 4 mm end diastolic fluid accumulation on cine images [23]. Presence and location of focal scars and myocardial fat infiltration were assessed visually with LGE and fat/water image analysis by both readers independently. In cases of uncertainty, a consensus read by a third reader (JSM, > 25 years of CMR experience) was conducted. Inter-observer comparison was based on a Bland–Altman analysis of the first 17 cases, evaluated separately by MF and EA.

### Statistical analysis

Normal distribution was assessed using the Shapiro–Wilk test. Continuous variables were compared between the three groups using either the Kruskal–Wallis method or one-way ANOVA followed by Dunn’s or Bonferroni’s post hoc tests. Comparisons between active and remission groups were made using Student’s *t*-test or Mann–Whitney *U* test for continuous variables. To evaluate the effect of different factors on T1, ECV, LV-strain and T2 values in patients with IBD, multivariate linear regression models with forward inclusion were used. The following fixed factors were included: age, sex, activity index, IBD subtype, mesalazine, azathioprine, monoclonal antibody treatment, and time since initial diagnosis. Categorical variables were compared using chi-squared or Fisher’s exact test. A significance level of 5% was considered statistically significant. Power analysis was not performed due to missing CMR data on this topic. Statistical calculations and graphs were performed using Matlab R2021b, Update 3 (The MathWorks, Inc) and SPSS, version 29. The graphical abstract was created using Biorender.com.

## Results

### Baseline characteristics

Initially, forty-six IBD patients were recruited. Two patients had to be excluded due to non-diagnostic CMR image quality caused by arrhythmias. Eighty-eight participants (26 with A-IBD, 18 R-IBD, 44 HV) were included in the final analysis. One patient with active disease declined administration of contrast agent and underwent native imaging. IBD-patients were well matched with control subjects for age, sex, and BMI. Patients in active and remission stage did not differ in terms of age, sex, subtypes of IBD, BMI, comorbidities, or cardiac related symptom history. See Table 1 for baseline characteristics. In accordance with the group classification, patients with active disease had higher clinical disease activity scores compared to patients in remission. Patients with active disease had a shorter disease duration and received systemic steroids more frequently. Six patients with active IBD received their initial diagnosis during the current hospitalization. There was no difference regarding other medical therapies between the two disease groups. Detailed information on medical therapy is provided in Table 2.

**Table 1:** Baseline characteristics of the Study Population

	All patients (N = 44)	Control group (N = 44)	Active (N = 26)	Remission (N = 18)	P^a^
Demographic features and comorbidity					
Male	20 (45)	20 (45)	10 (38)	10 (56)	0.26
Age, years	39.50 (31.50- 58.00)	38.50 (30.50- 53.50)	38.50 (27.00–56.00	42.00 (35.00–62.00	0.48
SBP, mmHg	118 ± 19	124 ± 16	115 ± 19	123 ± 17	0.124
DBP, mmHg	68 ± 11	75 ± 9	67 ± 12	70 ± 8	0.018
BMI, kg/m^2^	24.22 (20.45–26.59)	24.05 (21.57–26.22)	23.48 (20.08–26.78	24.87 (20.45–26.37	0.99
Diabetes mellitus	1 (2)	–	0	1 (6)	–
Arterial Hypertension	7 (16)	–	3 (12)	4 (22)	0.42^**b**^
Hyperlipidemia	6 (14)	–	3 (12)	3 (17)	0.68^**b**^
Disease type and activity					
Crohn ‘s Disease	30 (68)	–	17 (65)	13 (72)	0.75^**b**^
Ulcerative colitis	12 (27)	–	8 (31)	4 (22)	0.73^**b**^
Indeterminate colitis	2 (5)	–	1 (4)	1 (6)	–
CDAI, points	195 (117–364)	–	343 (217–508)	107 (77–168)	< .01^**b**^
SCCAI, points	7 (2–10)	–	10 (7–12)	2 (1 -2)	< .01^**b**^
Days since initial diagnosis	1436 (151–6542)	–	587 (69–5237)	2958 (1282 -7743)	< .01^**b**^
Laboratory results					
CRP, mg/l	2.72 ± 3.44	–	3.23 ± 3.66	1.85 ± 2.91	0.23
Hemoglobin, g/dl	12.63 ± 2.30	–	11.77 ± 2.30	13.94 ± 1.60	< .01
Hematocrit, %	37.60 ± 6.35	–	35.27 ± 6.62	41.18 ± 3.86	< .01
Albumin, g/dl	3.42 ± 0.74	–	3.16 ± 0.71	4.01 ± 0.37	< .01
Glomerular filtration rate, ml/min	99.23 ± 26.19	–	101.19 ± 26.80	96.24 ± 25.74	.57

*BMI *body mass index, *CDAI* Crohn’s disease activity index, *DBP* diastolic bloo pressure, *SBP* systolic blood pressure, *SCCAI* simple clinical colitis activity index

Values are n (%) or as otherwise stated, mean ± SD, or median (IQR), ^a^ Comparison between control, active and remission was performed using the ANOVA or Kruskal–Wallis test for continuous variables or chi-square test for categorical variables, ^b^ Comparison between active and remission was performed using the students t-test or Mann–Whitney-U test for continuous variables and chi-square or fishers exact test for categorical variables

**Table 2:** Medical therapy and symptom history

	All patients (n = 44)	Control group (n = 44)	Active (n = 26)	Remission (n = 18)	P^a^
Medical therapy					
Topical Steroids	3 (7)	–	2 (8)	1 (6)	1
Systemic Steroids	22 (50)	–	20 (77)	2 (11)	< .01
Mesalazine	10 (23)	–	7 (27)	3 (17)	.49
Azathioprin	3 (7)	–	2 (8)	1 (6)	1
Monoclonal antibody	17 (39)	–	7 (7)	10 (56)	.06
Symptom history					
Fatigue	33 (75)	0	20 (76.92)	13 (72.22)	.72
NYHA I	28 (63.64)	0	17 (65.38)	11 (61.11)	.53
NYHA II	8 (18.18)	0	4 (15.38)	4 (22.22)	.70
NYHA III	7 (7 (15.91)	0	5 (19.23)	2 (11.11)	.68
NYHA IV	1 (2.27)	0	0 (0)	1 (5.56)	/
Chest Pain	10 (22.73)	0	7 (26.92)	3 (16.67)	.48
Palpitation	18 (40.91)	0	11 (42.31)	7 (38.89)	.82
Previous Syncope	11 (25.00)	0	5	6	.31

*NYHA* New York Heart Association Functional Classification

Values indicate number (percentage), * comparison between active and remission cohort, categorial variables were compared using chi square or fishers exact test as appropriate.

### Symptom history

Thirty IBD patients (30/44, 68.12%) reported at least one symptom of heart failure (i.e., palpitations, dyspnea, or angina pectoris) with similar frequency in patients with A-IBD and R-IBD (18/26 vs. 12/18, p = 0.86). 16/44 (16.4%) described dyspnea on exertion and 10/44 (22.7%) reported occasional chest pain. See Table 2 for data on symptom history.

### Left and right ventricular function and dimensions

**Fig. 2:**
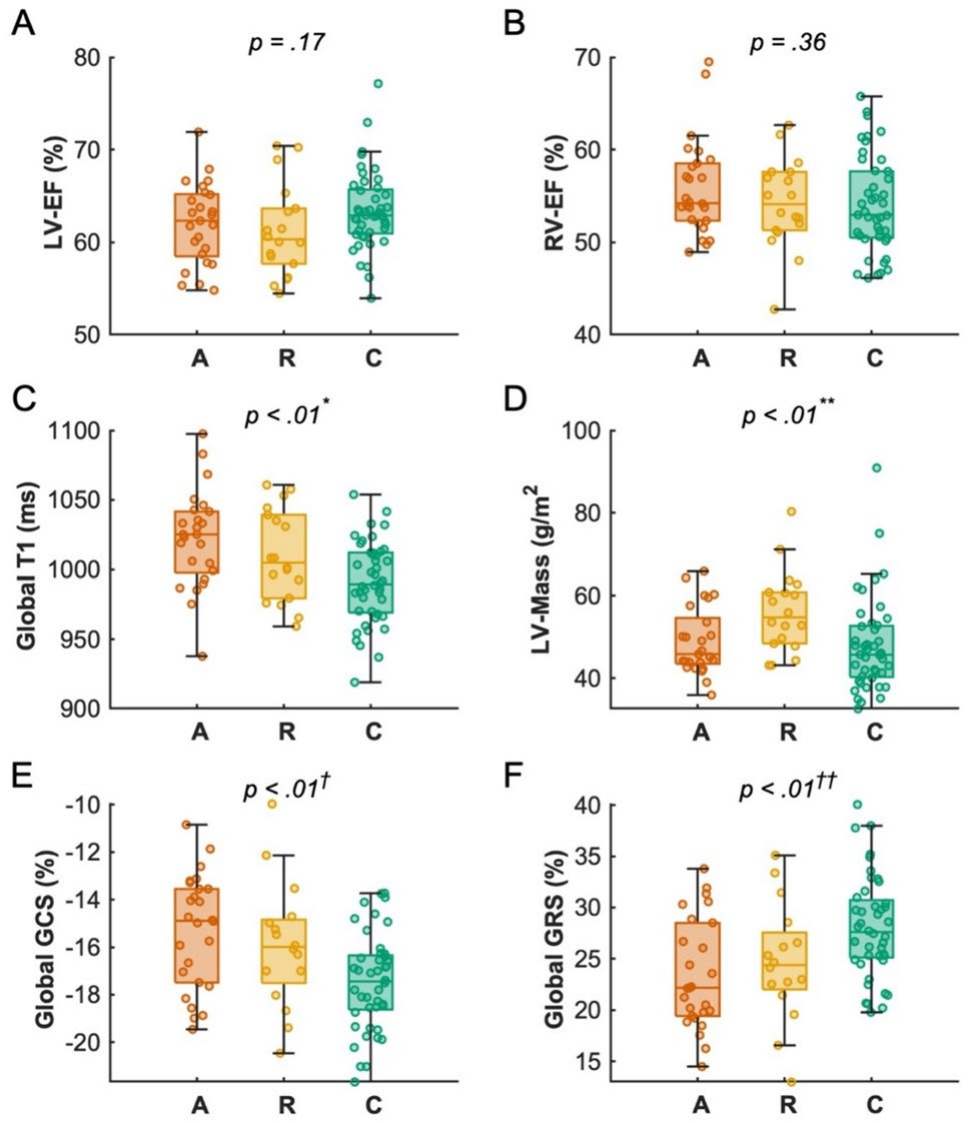
Comparison of Cardiac MR derived markers of reverse myocardial remodelling in patients with inflammatory bowel disease in Active (”A”) and Remission (“R”) stages of disease with healthy control group (“C”). Left (A) and right (B) ventricular ejection fraction; C) Cardiac MR derived myocardial marker of diffuse fibrosis and inflammation (Global T1), * post-hoc testing, active vs. control: p < .01, D) Left ventricular myocardial mass, ** remission vs. control: p = .02, E) Global circumferential strain (%) and F) Global radial strain (%) as marker of left ventricular dysfunction, † post-hoc testing, active vs. control: p < .01; †† active vs. control: p < .01; Boxplots indicate median plus interquartile range (IQR) and whiskers connect the upper or lower quartile to the nonoutlier maximum or minimum value, outliers are defined as > 1.5 * IQR

There was no significant difference in LV or RV sizes, stroke volumes or ejection fractions between active and remission IBD-patients and HV. Patients in remission had a significantly higher myocardial mass index compared to HV. See Table 3 and Fig. 2 for CMR results.

**Table 3:** CMR findings

	All patients (n = 44)	Control group (n = 44)	Active (n = 26)	Remission (n = 18)	P^b^
LGE presence	7/43^a^ (16)	–	3 (12)	4 (22)	.43
Non-ischemic LGE	7/43^a^ (16)	–	3 (12)	4 (22)	.43
Fat presence	5/44 (11)	–	4 (15)	1 (6)	.63
LV-EF, %	61.67 ± 4.58	63.41 ± 4.26	61.98 ± 4.36	61.23 ± 4.96	.17
LV-EDV indexed to BSA, ml/m^2^	79.56 ± 16.43	80.49 ± 18.27	75.73 ± 15.88	84.87 ± 16.10	.23
LV-Mass indexed to BSA, g/m^2^	49.97 (44.09–59.89)	45.73 (40.24–52.66)	45.78 (43.51–54.61)	54.76 (48.43–60.78)	< .01/ < .01
LA biplane area, cm^2^	19.63 (17.29–22.84)	21.05 (18.72–22.70)	19.40 (17.00–22.03	19.88 (18.15–23.55	.31
RV-EF	55.17 ± 5.03	54.06 ± 5.28	55.83 ± 5.14	54.21 ± 4.84	.36
RV-EDV indexed to BSA, ml/m^2^	86.37 (74.50–99.58)	87.26 (77.86–101.84	86.37 (75.51–95.63	86.42 (73.50–99.64	.64
Right atrium area, cm^2^	20.26 ± 4.16	20.86 ± 3.17	19.78 ± 3.88	20.95 ± 4.56	.44
Average basal T1, ms	1019.13 ± 34.24	995.73 ± 26.24	1022.44 ± 33.49	1014.54 ± 35.71	< .01 / < .01
Average medial T1, ms	1018.26 ± 38.58	992.11 ± 29.18	1022.71 ± 40.64	1011.72 ± 35.50	< .01/ < .01
Apical T1, ms	1010.33 ± 52.74	980.23 ± 47.85	1024.53 ± 49.91	987.61 ± 50.60	< .01 / < .01
Average basal T2, ms	48.63 ± 2.31	48.78 ± 2.44	48.75 ± 2.53	48.45 ± 2.01	.88
Average medial T2, ms	49.03 ± 2.36	49.33 ± 2.58	49.25 ± 2.64	48.70 ± 1.91	.65
Apical T2, ms	49.81 ± 2.64	49.88 ± 2.86	50.26 ± 2.78	49.12 ± 2.31	.41
Basal ECV, %	22.87 ± 2.79	–	23.41 ± 2.36	22.07 ± 3.26	.17
Medial ECV, %	23.40 ± 2.97	–	24.14 ± 2.68	22.20 ± 3.13	.06
Apical ECV %	25.46 ± 4.25	–	26.17 ± 4.71	24.42 ± 3.40	.30

*BSA* body surface area, *ECV* extracellular volume, EDV end diastolic volume, *EF* ejection fraction, *LV* left ventricular, *RV* right ventricular; *SV* stroke volume

Numbers indicate n (%) or as otherwise stated, ^a^ One patient declined contrast agent application, ^b^ Comparison between control, active and remission was performed using the ANOVA or Kruskal–Wallis test for continuous variables or chi-square test for categorical variables, in case of P < 0.05 followed by post-hoc testing, all second values indicate active vs. control, other tests were not significant.

### Left ventricular systolic function: strain analysis

Patients with active disease but not in remission had significantly impaired GRS and GCS values compared to HV (GRS: Active: 23.40% ± 5.34%, Remission: 24.64% ± 5.78%, HV: 28.06% ± 4.92%; p < 0.01; GCS:—15.31% ± 2.38% vs.—15.93% ± 2.65% vs.—17.48% ± 2.02%, p < 0.01). Neither age, sex, type of medical treatment, IBD subtype, activity index nor time since initial diagnosis was associated with GLS, GRS or GCS (all p > 0.05).

### Symptom history and correlation with cardiac function

Patients who reported symptoms of heart failure had impaired systolic ventricular function when compared with IBD patients that did not report cardiac symptoms.: GCS: -15.00% vs. -16.77%,p = 0.03 and GRS: 22.69% vs. 26.50%, p = 0.04.

### Markers of diffuse myocardial fibrosis: T1 mapping and ECV

After applying exclusion criteria, 1271 of 1408 myocardial segments (90.3%) were eligible for final T1 analysis. Patients with active disease had higher native global T1 values compared to HV (Active 1022.0 ± 34.83 ms, Remission: 1010.10 ± 32.88 ms, HV: 990.61 ± 29.35, P < 0.01 between active and HV, other NS).

**Fig. 3:**
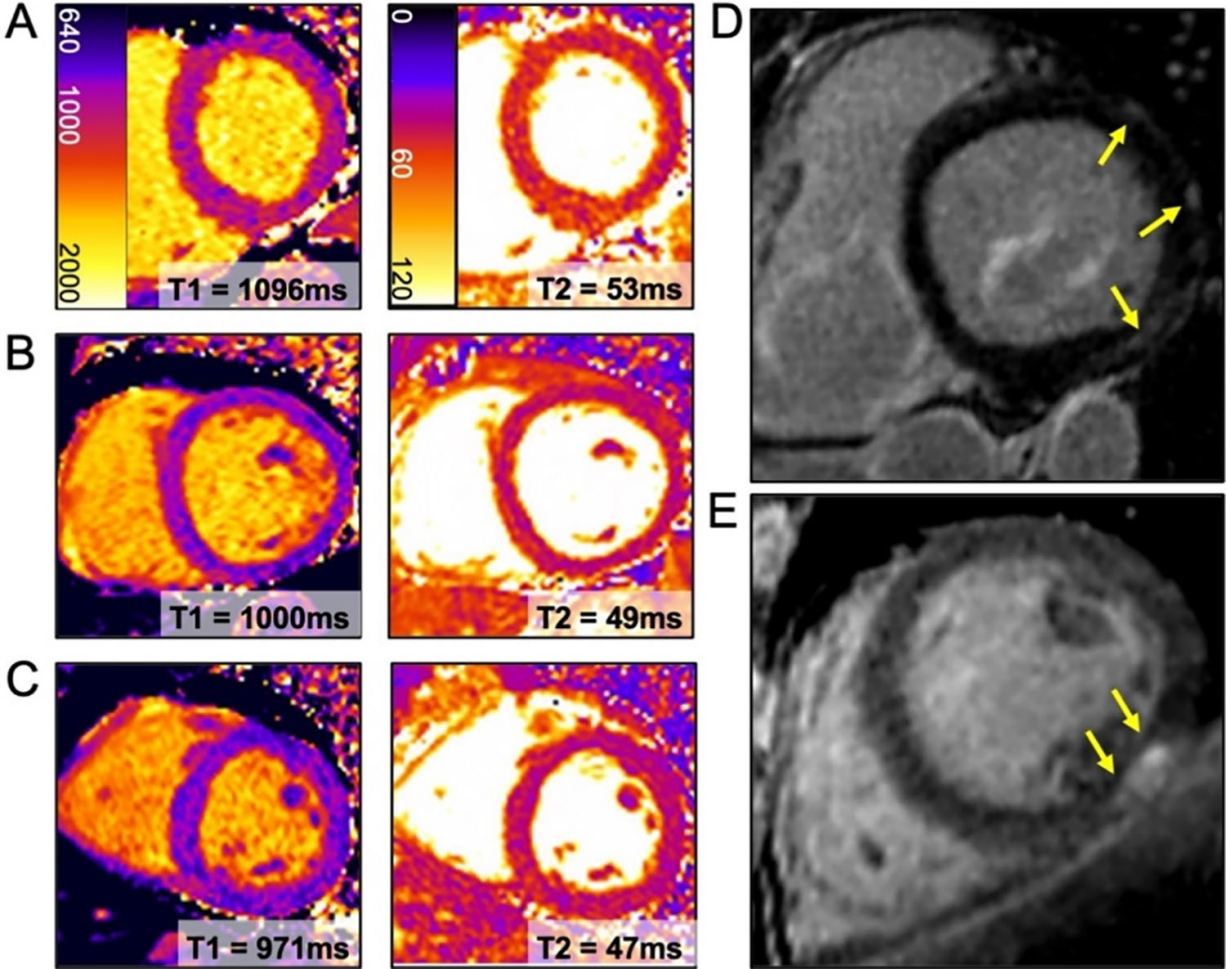
Representative Examples of cardiac MRI derived markers of diffuse fibrosis (T1 mapping) and edema (T2 mapping) (A-C) and focal fibrosis (late gadolinium enhancement) (D + E); **A**: Male patient with active ulcerative colitis and elevated markers of fibrosis and inflammation **B**: Male patient with Crohn’s disease in remission **C**: Male Healthy volunteer **D**-**E**: Non-ischemic scar (yellow arrows, areas of high signal intensity) in patients with Crohn’s disease (D) and Ulcerative colitis (E)

At global and slice levels, ECV values did not differ between patients with active disease and in remission. In a multivariate linear regression model, global T1 and ECV correlated with disease activity (T1: r = 0.33, p = 0.02; ECV: r = 0.27, p = 0.05), whereas age, sex, IBD subtype, mesalazine, azathioprin, monoclonal antibody treatment, myocardial global strain values and time since initial diagnosis were not significantly correlated with global T1 or ECV values (all p > 0.05). Examples of CMR findings are shown in Fig. 3.

### Myocardial inflammation

**Fig. 4:**
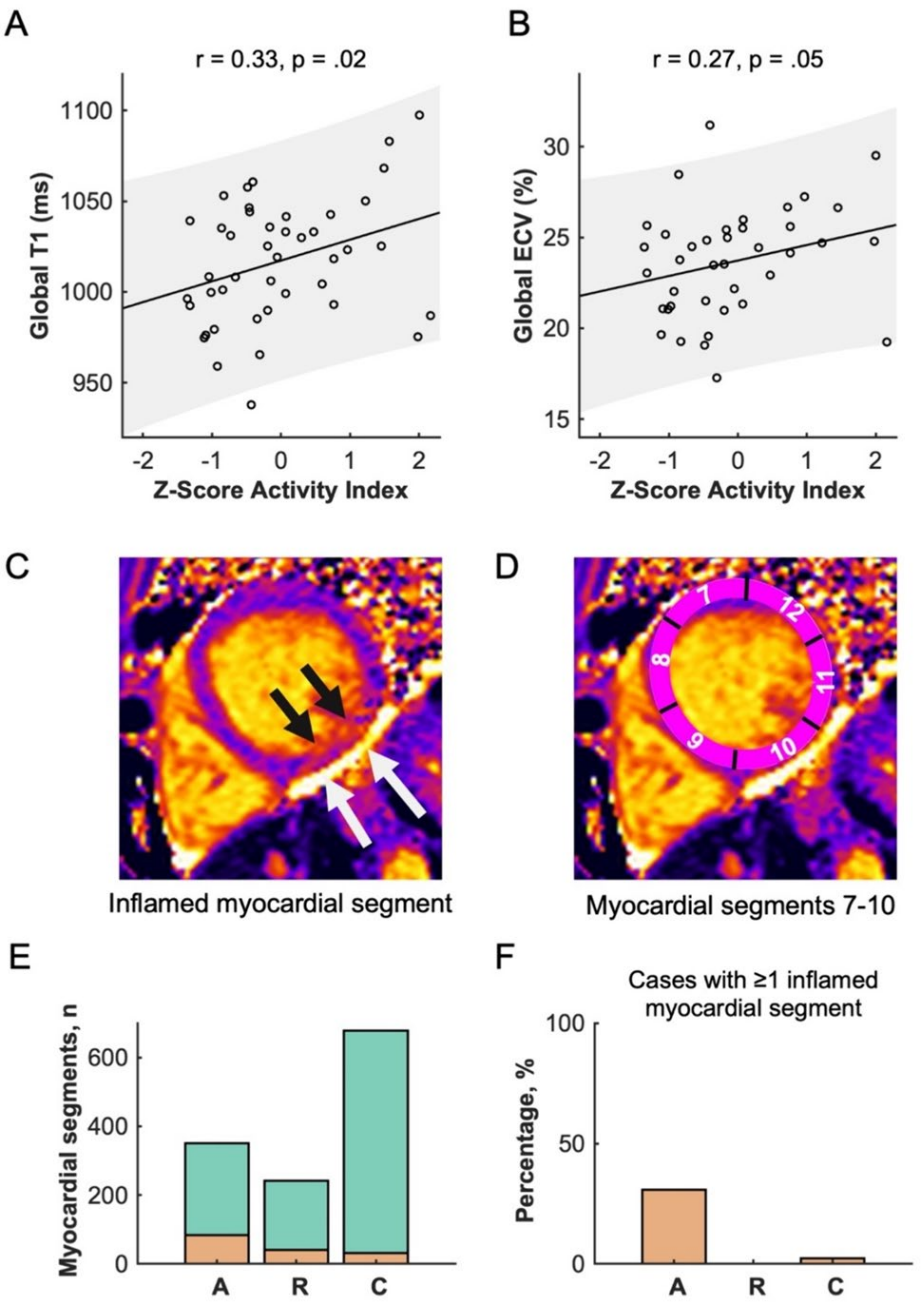
Disease activity in patients with inflammatory bowel disease in correlation with cardiac MR markers of diffuse fibrosis. (A) Global T1 and (B) global Extracellular volume (ECV). Grey area = 95% Confidence Bounds, Z-Score Activity Index = (Score of the Crohn’s disease activity index [CDA] or Simple Clinical Colitis Activity Index [SCCAI]) – mean (CDAI or SCCAI) / standard deviation (CDAI or SCCAI), higher values indicating more severe disease activity; **C** Midventricular native T1 map, focal T1 elevation: 1134 ms, cut-off: > 1049.3 ms (black arrows) and pericardial effusion (white arrows) **D** Schematic division of the myocardium according to the American heart association model, inflammation mainly found in segment 10 **E** Absolute number of analyzed myocardial segments with normal T1 values (green) and elevated T1 values (red) **F** Percentage of cases with 1 ≥ inflamed myocardial segment;”A” active disease, “R” remission, “C” healthy contro

In A-IBD 83/351 (23.6%), in R-IBD 40/242 (16.5%) and in HV 31/678 (4.6%) myocardial segments had prolonged native T1 times. Significantly more participants with active disease (8/26, 30.8%) had at least one abnormal T1 and T2 segment in the same AHA-position than patients in remission (0/18) or HV (1/44, 2.3%, P < 0.01). The number of coincident abnormal T1 and T2 segments in the same left ventricular myocardial-position were distributed as follows: A-IBD 20/350 (5.7%), R-IBD 0/238 (0%), HV 1/678 (0.4%), see Fig. 4.

### Focal myocardial fibrosis (LGE imaging) and myocardial fat infiltration

In forty-three IBD-patients LGE imaging and in forty-four fat imaging was available for analysis. A positive LGE finding in a non-ischemic pattern was present in 7/43 patients (16.3%). T2 values did not exceed the upper local reference limit in any of the AHA-segments with a positive LGE finding. LGE in an ischemic pattern was not present in any of the patients. There was no difference (p = 0.43) in the presence of LGE between patients with active disease (3/24, 12%) and in remission (4/18, 22.2%, p = 0.43). Myocardial fat infiltration was detected in 5/44 (11.4%) patients with no difference (p = 0.63) between active and remission IBD. LGE and fat infiltration were not associated with the use of monoclonal antibody therapy (p = 0.685 for LGE and p = 1 for fat infiltration).

### Association of arterial hypertension with LV fibrosis and markers of LV remodeling

There were no differences in LV-mass, LV-function, and LV strain markers between IBD-patients with and IBD patients without hypertension. Please see table S1 in the supplement for detailed results. Furthermore, T1 and ECV mapping showed no differences in markers of diffuse fibrosis between these groups. However, hypertension was significantly associated with LGE presence (p = .001).

### Marker of global and slice level edema: T2 mapping

After exclusion, 1397 of 1408 myocardial segments (99.2%) were eligible for final T2 analysis. Patients with active disease and in remission as well as HV did not differ regarding T2 values on a global and slice level (Active disease: T2 global 49.33 ± 2.37, Remission: 48.67 ± 1.85, HV: 49.27 ± 2.50; p = 0.61). In patients with IBD global T2 values did not correlate with age, sex, activity index, IBD subtype, mesalazine, azathioprin, monoclonal antibody treatment, myocardial global strain values and time since initial diagnosis. The number of AHA segments with T2 values below the lower reference limit (44.28 ms) and the number of participants with at least one lower T2 segment did not differ between patients with active disease and in remission as well as HV (p = .184, p = .410 respectively).

### Pericardial effusion

Nineteen IBD patients (19/44, 43.2%) had pericardial effusion. A-IBD patients (13/26, 50%) were more likely to have pericardial effusion then R-IBD (6/18, 33.3%) and healthy volunteers (8/44, 18.2%; P < .01). Patients with presence of pericardial effusion had significantly more inflamed myocardial segments then patients without pericardial effusion (15/277, 5.4% vs. 5/311, 1.6%, p = .01).

### Inter-reader Comparison

There was excellent agreement between the two readers. The average differences ± the standard deviation of the differences between both readers were as follows: LV-EF: −2.3 ± 2.9%; RV-EF: −1.4 ± 2.9%, LVEDV: -2.1 ± 6.7 ml, RVEDV: 2.9 ± 12.2 ml, T1: −1.1 ± 11.4 ms, T2: -0.0 ± 0.4 ms.

## Discussion

This study provides evidence for myocardial involvement and patterns of adverse left ventricular remodeling in patients with IBD. Using CMR-derived myocardial tissue phenotyping, myocardial involvement and adverse left ventricular remodeling can already be detected in IBD-patients with active disease but without diagnosis of heart failure or inflammatory heart disease. Adverse cardiac remodeling was characterized by subclinical impaired left ventricular function, low-grade myocardial inflammation, diffuse as well as focal fibrosis accumulation and pericardial effusion.

### IBD, inflammatory heart disease and adverse cardiac remodeling

Population based studies have shown an association between IBD-patients and an increased risk of cardiovascular death and heart failure, especially during periods of active disease [4, 5]. The underlying mechanisms are incompletely understood. Cardiovascular disease in IBD patients has been linked to chronic systemic inflammation rather than to traditional risk factors [6]. Clinically manifest peri-myocarditis as a rare extra-intestinal manifestation of inflammatory bowel disease has been described in case reports [24–26]. Cases were characterized by chest pain and segmentally elevated myocardial T1 and T2 values as well as LGE in a non-ischemic pattern on CMR examinations. Findings resolved after adequate treatment of IBD. Using CMR as the gold-standard for myocardial tissue characterization in suspected cardiomyopathy [27], the results of this study hopefully add to these observations.

### Symptoms of heart failure and left ventricular dysfunction

The majority of IBD patients in this study, which excluded patients with known cardiovascular disease, reported at least one symptom related to heart failure, with 16/44 (16.4%) describing dyspnea on exertion and 10/44 (22.7%) reporting occasional chest pain. A-IBD patients had evidence of subclinical left ventricular dysfunction as indicated by impaired left ventricular strain markers and IBD patients who reported heart failure-related symptoms had impaired systolic function on left ventricular strain analysis compared with IBD patients without symptoms. Several echocardiographic studies already have demonstrated subclinical systolic functional impairment in patients with IBD, measured with GLS or GCS [28–30]. In patients with CD, the extent of functional impairment correlated strongly with the disease’s activity index [29]. We hypothesize that subclinical inflammation and fibrosis accumulation may be underlying mechanisms that contribute to the observed impaired systolic function and warrants further investigation.

### Elevated CMR markers of fibrosis and inflammation

A-IBD patients had elevated global T1 values as a marker of diffuse fibrosis. In an UK biobank study, which studied the association of native T1 with incident clinical events in 42,308 participants, elevated native T1 was associated with a risk of incident heart failure and all-cause mortality [31]. Furthermore, 30.8% of A-IBD patients in our study had at least one myocardial segment with concomitant elevated T1 and T2 values, indicating non-ischemic inflammation [10]. In line with this, scarring and focal fibrosis in a strictly non-ischemic pattern was found in 16.3% of all IBD patients on LGE imaging. As the presence of LGE predicts adverse cardiac outcomes in patients with non-ischemic cardiomyopathy [32], the influence on outcome in patients with IBD should be investigated by further research. Cardiovascular imaging, especially CMR, plays a crucial role in assessing the extent of cardiac involvement across different systemic immune-mediated diseases and allows identification of cardiac involvement even in sub clinical stages [9]. Ntusi et al. found subclinical cardiovascular disease in patients with rheumatoid arthritis, including focal and diffuse myocardial fibrosis and inflammation and impaired myocardial strain [33]. Findings that resemble those observed in this study.

### Arterial Hypertension and association with CMR derived markers of left ventricular remodeling

In this study, patients with arterial hypertension were associated with LGE presence. This results needs to be put into context: Left ventricular adaption to systemic hypertension includes LV-wall thickening, LV-mass increase and myocardial fibrosis deposition[34]. The two major types of myocardial fibrosis are replacement, known as focal fibrosis and reactive, known as diffuse fibrosis [35]. Although both forms can coexist, diffuse fibrosis is the characteristic pattern in patients with hypertensive heart disease [34]. In terms of CMR imaging modalities, LGE is able to show focal myocardial fibrosis while T1 and ECV mapping is the imaging gold standard for detecting diffuse fibrosis [36, 37]. Except for the presence of LGE, IBD patients with hypertension in this study did not differ significantly from patients without hypertension in terms of CMR markers of structural and functional left ventricular remodeling. Most importantly, markers of diffuse myocardial fibrosis were not elevated in patients with hypertension. Thus, focal myocardial fibrosis as a direct consequence of hypertension does not seem to be the most obvious explanation, although it is possible [38]. Rather, we hypothesize that the presence of hypertension may modulate the extent of systemic inflammation-induced myocardial damage as indicated by LGE imaging.

### IBD and the risk of ischemic heart disease

While a recent meta-analysis demonstrated an increased risk for coronary artery disease (CAD) in IBD-patients [39], this study did not reveal subclinical ischemic scarring, i.e. subendocardial hyperenhancement on LGE imaging, in a relatively young IBD cohort with few traditional cardiovascular risk factors. However, presence of CAD cannot be ruled out in this study with certainty because no anatomical tests such as coronary computed tomography angiography (CTA) were performed. Risk estimation systems such as the Systematic COronary Risk Evaluation (SCORE) score, could be applied to estimate the 10-year risk of fatal cardiovascular disease. However, as discussed previously [40], these scores do not reflect the presence of systemic chronic inflammation and current changes in cholesterol or blood pressure may be caused by IBD disease flares. Furthermore, overlapping risk factors, inflammation, microbiome abnormalities, endothelial dysfunction, thrombogenicity, lipid dysfunction, and the impact of corticosteroids may promote the development of CAD in IBD-patients [8], as may be seen in larger cohorts. The detection of CAD using e.g. coronary CTA is therefore warranted and should be considered in a follow-up study.

### Drug treatment as a possible contributor to myocardial alterations

Cases of mesalazine and monoclonal antibody induced (peri)-myocarditis have been reported as a rare entity [41, 42]. However, this study did not find an association between global T1, T2, ECV values or LGE and mesalazine or monoclonal antibody treatment, although the sample size may not have been sufficiently large to study this entity. Left ventricular mass was elevated in patients in remission. Treatment with systemic steroids is used in patients with IBD to induce symptomatic remission [1] and high cortisol levels have previously been associated with increased left ventricular mass index (LVM-I) [43]. Patients in remission had a significantly longer disease duration compared to patients with active disease. It is therefore possible that patients in remission were exposed to higher cumulative steroid doses over time, which may have led to increased LVM-I. However, due to long-term use and dose variations in response to changes in disease activity, information on steroids is often inaccurate and incompletely recorded in patients’ medical records [44]. The assessment of cumulative steroid dosage is further complicated by the fact that patients are often treated in an ambulant setting, sometimes receive different substances and data exchange is commonly omitted between different patient management systems. Nevertheless, efforts should be made in a follow-up study to estimate the cumulative steroid dose, e.g. prospectively using a glucocorticoid cumulative dose calculator [44].

### Gut-heart axis interaction

To explore the pathophysiological mechanisms of this study’s findings, the interaction between the gut and the heart should be further investigated. Loss of intestinal barrier function is important in the pathogenesis and disease course of IBD [45, 46]. Subsequent translocation of bacterial lipopolysaccharides from the intestinal to the circulation system may cause myocardial damage [47]. The relationship between heart failure and altered intestinal morphology and function has also been recognized [48]. Consequently, studying the relation between loss of gut barrier function, microbiome abnormalities, altered cardiac function, and myocardial tissue composition is likely to yield further insight into the concomitant presence of IBD and adverse cardiac remodeling.

### Implications for clinical practice

Using CMR this study demonstrates subclinical myocardial involvement in patients with active inflammatory bowel disease. These findings underline the recommendation that prevention, systematic detection, and aggressive management of cardiovascular risk factors should constitute components of management for IBD-patients as well as maintaining long-term remission in patients with IBD to minimize the risk of inflammation-induced cardiovascular events [7].

## Limitations

This observational study primarily focused on the use of CMR to detect subclinical myocardial involvement in patients with IBD and was designed to inform further research hypotheses. However, there are limitations to consider. Participants in this study were recruited from a single center only. Additionally, no repeat endoscopy before the CMR scan was performed in patients in remission due to ethical constraints. Consequently, patients with clinically inapparent but endoscopically detectable bowel inflammation may have been included as patients in remission, potentially leading to referral bias in our study. Secondly, we carefully excluded patients with known cardiovascular disease. However, we did not use invasive or non-invasive testing to assess CAD. The detection of CAD using methods such as coronary CTA is therefore warranted and should be considered in a follow-up study. Finally, we did not assess blood markers of myocardial injury and dysfunction. Hence, high-sensitivity troponin or NT-pro-BNP levels were not available in this study. Correlating blood markers of myocardial injury and dysfunction with imaging markers of adverse myocardial remodeling, inflammation, and damage may improve our understanding of cardiac involvement in IBD patients. These assessments should be included in a larger study based on the results of this work.

## Supplementary Information

Below is the link to the electronic supplementary material.

Supplementary file1 (DOCX 17 kb)

## Data Availability

The data that support the findings of this study are available from the corresponding author upon reasonable request.

## Abbreviations

CD: Crohn’s disease
CDAI: Crohn’s disease activity index
ECG: Electrocardiogram
GCS: Global circumferential strain
GLS: Global longitudinal strain
GRS: Global radial strain
HV: Healthy volunteers
IBD: Inflammatory bowel disease
LGE: Late gadolinium enhancement
SCCAI: Simple clinical colitis activity index
UC: Ulcerative Colitis
