# Supplementary material for: Cardiovascular magnetic resonance reveals myocardial involvement in patients with active stage of inflammatory bowel disease
Source: Clin Res Cardiol. 2024 Aug 5;114(9):1164–75. doi: 10.1007/s00392-024-02503-5 (PMC12408709; doi:10.1007/s00392-024-02503-5)
Supplement: Supplementary file 1 — Supplementary file1 (DOCX 17 kb) [file 392_2024_2503_MOESM1_ESM.docx]

**Cardiovascular magnetic resonance reveals myocardial involvement in patients with active stage of inflammatory bowel disease**

Maximilian Fenski^1^, Endri Abazi^,1^, Jan Gröschel^1^, Thomas Hadler^1^, Diane Kappelmayer^2^, Frank Kolligs^2^, Claudia Prieto^3,6^, Rene Botnar^3,6,7^, Karl-Philipp Kunze^3,4^, Jeanette Schulz-Menger^1,5^

1 Working Group Cardiovascular Magnetic Resonance, Experimental and Clinical Research Center, Charité Medical Faculty, Max-Delbrück Center for Molecular Medicine, HELIOS Klinikum Berlin Buch, Department of Cardiology and Nephrology, Berlin, Germany

2 HELIOS Klinikum Berlin Buch, Department of Internal medicine and Gastroenterology, Berlin, Germany

3 School of Biomedical Engineering and Imaging Sciences, King’s College London, London, UK

4 MR Research Collaborations, Siemens Healthcare Limited, Camberley, UK

5 DZHK (German Center for Cardiovascular Research), Partner Site Berlin

6 School of Engineering, Pontificia Universidad Católica de Chile, Santiago, Chile

7 Institute for Biological and Medical Engineering, Pontificia Universidad Católica de Chile, Santiago, Chile

Corresponding author

Prof. Dr. Jeanette Schulz-Menger

Experimental and Clinical Research Center (ECRC)

Charité – Universitätsmedizin Berlin

Lindenberger Weg 80, 13125 Berlin, Germany

Email: jeanette.schulz-menger@charite.de

**Sequence parameters**

**Late gadolinium enhancement:**

3D LGE Dixon Fat/Water imaging, inversion recovery prepared spoiled gradient-echo prototype sequence (SIEMENS WIP 1111), ECG-gated in free-breathing and transversal orientation, isotropic resolution of 1.3mm^3^, image navigator placed manually directly over the left ventricle. Acquisition parameters were as follows: TE1= 1.31ms; TE2= 2.81ms; FA 20°,. TI individually determined using a TI-Scout (SIEMENS WIP 1090). To account for the time delay during image acquisition mean of 20 ms was added. Trigger delay (TD) and acquisition window were selected based on an automatic resting phase detection, based on CINE-images acquired in 4CV directly before 3D data acquisition.

2D: PSIR reconstruction, gradient echo sequences, TI 240ms, TR 29.76ms, TE 5.17ms, FA 30°, FOV 350x263mm^2^, matrix 256x166, voxel size 1.4x1.4mm^2^, ST 7mm, gap between slices 0mm.

**Balanced steady-state free precession cine imaging:**

**Long** axis: ECG triggered with retrogating, repetition time (TR) 2.78 ms, 30 reconstructed phases, echo time (TE) 1.19ms, field of view (FOV) 340x276mm^2^, matrix 192x156, voxel size 1.8x1.8mm^2^, slice thickness (ST) 6mm, flip angle (FA) 74°, GRAPPA acceleration factor 2.

**Short axis:** ECG triggered with retrogating, TR 3.31ms, 30 reconstructed phases, TE 1.44ms, FOV 380x308.75mm^2^, matrix 192x156, voxel size 2.0x2.0mm^2^, ST 7mm, gap between slice: 0mm, FA 80°, GRAPPA acceleration factor 2.

**T2 mapping:**

Balanced steady-state free precession sequence, T2prep times 0, 25 and 55 ms, 3 recovery heartbeats, TR 2.98ms, TE 1.12ms, FA 70°, FOV 380×288mm^2^, matrix 224x170, voxel size 1.7x1.7mm^2^, ST 8mm, gap between slices 0mm, GRAPPA acceleration factor 2, motion corrected.

**T1 Mapping:**

5(3)3 MOLLI acquisition scheme, TR 3.9ms, TE 1.13ms, FA 35°, TI 180ms, FOV 360×270mm2, matrix 256x144, voxel size 1.4x1.4mm^2^, ST 8 mm, gap between slices 0mm, GRAPPA acceleration factor 2, motion corrected.

**Synthetic Extracellular volume:**

Prototype-sequence,

Pre-contrast: 5(3)3 MOLLI acquisition scheme, TR 3.9ms, TE 1.13ms, FA 35°, TI 180ms, FOV 360×270mm2, matrix 256x144, voxel size 1.4x1.4mm^2^, ST 6 mm, GRAPPA acceleration factor 2, motion corrected.

Post-contrast: 4(1)3(1)2 MOLLI acquisition scheme, TR 5ms, TE 1.13ms, FA 35°, TI 260ms, FOV 360×270mm2, matrix 256x144, voxel size 1.4x1.4mm^2^, ST 6 mm, GRAPPA acceleration factor 2, motion corrected.

### Association of arterial hypertension with LV fibrosis and markers of LV remodeling

| Parameter | Hypertension (n = 7) | No Hypertension ( n= 37) | P |
| --- | --- | --- | --- |
| LV-mass index, g/m^2^ | 53.25 ± 10.91 | 51.64 ± 9.34 | .688 |
| LV-EF, % | 61.16 ± 4.52 | 61.77 ± 4.64 | .750 |
| T1 global, ms | 1000.97 ± 21.39 | 1020.12 ± 35.49 | .178 |
| T2 global, ms | 50.22 ± 1.95 | 48.84 ± 2.16 | .124 |
| ECV global, ms | 22.69 ± 3.09 | 23.82 ± 3.05 | .411 |
| GLS, % | -15.29 ± 5.55 | -17.78 ± 1.85 | .285 |
| GRS, % | 22.43 ± 6.08 | 24.11 ± 5.43 | .493 |
| GCS, % | -14.88 ± 2.79 | -15.66 ± 2.45 | .482 |
| LGE presence, n (%) | 4/7 (57.1) | 3/36 (8.3) | .001 |
| Fat presence, n (%) | 0/7 (0) | 4/37 (13.5) | 1 |

Table S1 Analysis of CMR results in Patients with IBD and Hypertension vs. IBD without Hypertension; ECV = extra cellular volume, GCS = global circumferential strain, GLS = global longitudinal strain, GRS = global radial strain
